# Supplementary material for: A Seasonal Study of Koi Herpesvirus and Koi Sleepy Disease Outbreaks in the United Kingdom in 2018 Using a Pond-Side Test
Source: Animals (Basel). 2021 Feb 9;11(2):459. doi: 10.3390/ani11020459 (PMC7916346; doi:10.3390/ani11020459)

Figure S1. Examples of common carp showing clinical signs of carp pox disease (A, B) and koi herpesvirus disease (C, D). *: Skin lesions (A, B) appearing as a thick mucus coat, caused by cyprinid herpesvirus (CyHV)-1 infection. Arrows: Gill necrosis (C) and skin ulceration (D) caused by CyHV-3 infection.

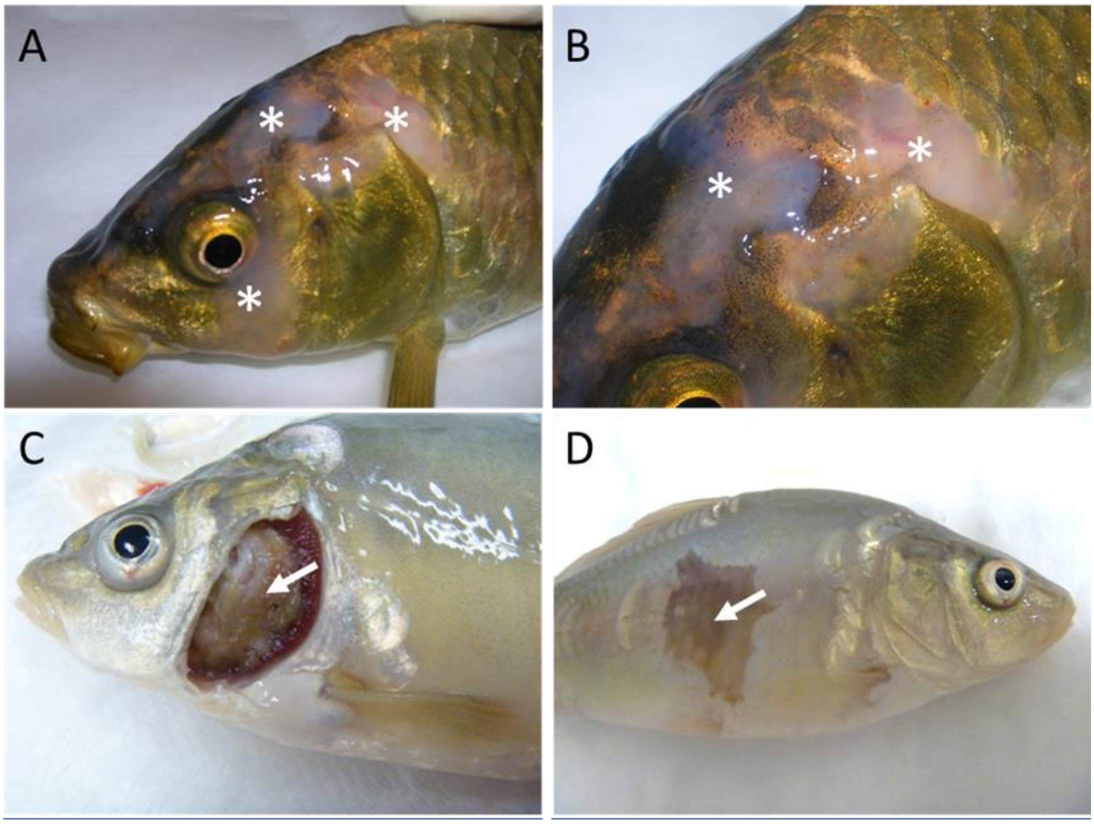

Supplement: Supplementary file 1 [file animals-11-00459-s001.zip › Figure S1.pdf]
