# Supplementary material for: A Seasonal Study of Koi Herpesvirus and Koi Sleepy Disease Outbreaks in the United Kingdom in 2018 Using a Pond-Side Test
Source: Animals (Basel). 2021 Feb 9;11(2):459. doi: 10.3390/ani11020459 (PMC7916346; doi:10.3390/ani11020459)

Supplement Figure S2. LAMP identification of KHV (CyHV-3) and CEV in common carp skin and gill swabs. The host *ef1a* LAMP assay was used as internal control. LAMP detection expressed as time of positivity Tp (minutes:seconds) and anneal temperature (°C). KHV detection by qPCR showing the cycle threshold (ct).

Site	Swab No.	DNA (ng/ul)	qPCR Ct	KHV LAMP Tp (mm:ss)	KHV LAMP Anneal (°C)	Carp ef1a LAMP Tp (mm:ss)	Carp ef1a LAMP Anneal (°C)	CEV LAMP Tp (mm:ss)	CEV LAMP Anneal (°C)
A	1	485.4	25.00	06:30	89.8	undetected	undetected	undetected	undetected
A	2	498.8	24.18	06:15	89.8	08:15	88.1	undetected	undetected
A	7	423.1	26.22	06:45	89.9	07:45	88.3	undetected	undetected
A	8	836.7	21.96	06:00	89.7	07:00	88.1	undetected	undetected
A	9	492.3	26.08	06:45	89.7	06:45	88.1	undetected	undetected
B	1	506.3	28.93	08:15	89.8	15:30	88.2	undetected	undetected
C	1	400.1	20.99	05:30	89.6	06:30	88.1	02:15	undetected
C	2	249.5	18.32	04:45	89.8	06:45	88.2	19:30	82.0
C	3	182	22.98	05:45	89.7	07:00	88.0	undetected	undetected
C	4	175.9	21.68	05:30	89.8	06:30	88.1	20:00	82.0
C	5	191.5	22.11	05:30	89.7	06:30	88.0	undetected	undetected
D	1	359	35.24	00:00	89.9	00:00	88.0	undetected	undetected
E	1	310	26.02	06:30	89.7	08:15	88.0	undetected	undetected
E	2	452	26.16	06:30	89.6	07:45	88.1	undetected	undetected
E	3	326	25.62	06:30	89.7	07:45	88.1	undetected	undetected
E	4	533.7	32.69	undetected	89.9	07:15	88.3	undetected	undetected
E	5	455	27.84	07:00	89.3	07:30	88.0	undetected	undetected
F	1	520.5	23.86	06:45	89.8	undetected	88.1	undetected	undetected
G	1	267.9	undetected	undetected	undetected	undetected	undetected	undetected	undetected
G	2	162.3	undetected	undetected	undetected	undetected	undetected	undetected	undetected
G	3	144.5	undetected	undetected	89.9	undetected	undetected	undetected	undetected
G	4	111.1	undetected	12:00	89.9	undetected	undetected	undetected	undetected
G	5	237.4	undetected	undetected	undetected	undetected	undetected	undetected	undetected
H	1	874.2	undetected	undetected	undetected	06:45	88.2	undetected	undetected
I	1	441.6	21.19	06:15	89.7	undetected	70.5	undetected	undetected
I	2	348.1	27.52	06:45	89.7	undetected	70.5	undetected	undetected
I	3	412.8	undetected	08:00	89.7	undetected	87.8	undetected	undetected
I	4	480.7	30.51	09:00	89.9	15:00	87.5	undetected	undetected
J	1	189	32.74	08:12	89.8	06:30	88.2	undetected	undetected
J	2	512.9	22.02	05:27	89.8	06:30	88.2	undetected	undetected
K	1	255.3	29.16	08:00	undetected	undetected	undetected	undetected	undetected
K	2	280.8	25.68	undetected	undetected	undetected	undetected	undetected	undetected
K	3	313.8	undetected	undetected	undetected	undetected	undetected	undetected	undetected
K	4	178.1	27.18	07:45	undetected	undetected	undetected	undetected	undetected
K	5	213.7	31.36	08:00	undetected	undetected	undetected	undetected	undetected
K	6	344.3	24.11	undetected	undetected	02:30	undetected	undetected	undetected
K	7	580.5	22.28	06:00	89.7	01:45	88.1	undetected	undetected
K	8	868.3	21.64	06:00	89.8	08:30	88.1	undetected	undetected
K	9	722	20.86	06:00	89.9	07:15	88.2	undetected	undetected
K	10	484.6	24.85	06:45	89.7	undetected	undetected	undetected	undetected
L	1	449.4	23.40	07:00	89.9	undetected	70.4	undetected	undetected
L	2	424.6	undetected	08:00	89.6	undetected	undetected	undetected	undetected
L	3	539.6	30.48	08:45	89.7	undetected	undetected	undetected	undetected
L	4	881.7	22.92	07:00	89.5	11:45	88.2	undetected	undetected
L	5	575.4	undetected	08:15	89.4	undetected	undetected	undetected	undetected
L	6	507.5	23.24	07:00	90	undetected	70.7	undetected	undetected
L	7	284.6	26.67	07:00	89.8	undetected	70.7	undetected	undetected
L	8	403.9	undetected	13:30	89.6	undetected	undetected	undetected	undetected
L	9	481.9	22.11	07:00	90	undetected	70.6	undetected	undetected
L	10	348.8	28.78	07:00	90	12:30	88.3	undetected	undetected
M	1	191.2	undetected	undetected	undetected	undetected	undetected	undetected	undetected
M	2	181.9	undetected	undetected	undetected	09:15	88.1	undetected	undetected
N	1	504.8	27.50	07:45	89.4	07:00	88.0	undetected	undetected
N	2	449.4	25.32	07:00	89.8	08:00	88.1	undetected	undetected
N	3	436.1	35.07	00:00	undetected	07:00	88.1	undetected	undetected
O	1	882.1	undetected	undetected	undetected	undetected	88.2	undetected	undetected
O	2	748.7	undetected	undetected	undetected	11:15	88.4	undetected	undetected
O	3	585.6	undetected	undetected	undetected	06:30	88.3	undetected	undetected
P	1	568.7	21.53	06:30	89.8	undetected	87.4	undetected	undetected
P	2	515.9	23.83	07:00	89.9	undetected	undetected	undetected	undetected
P	3	498.3	undetected	06:30	89.8	undetected	undetected	undetected	undetected
P	4	320.8	25.21	07:00	89.9	undetected	87.8	undetected	undetected
P	5	411.4	22.76	06:15	89.8	undetected	undetected	undetected	undetected

	Amplification	Amplification Rate	Anneal Derivative

A **KHV**

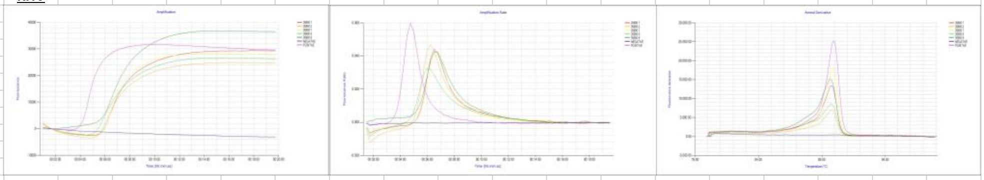

ef1a

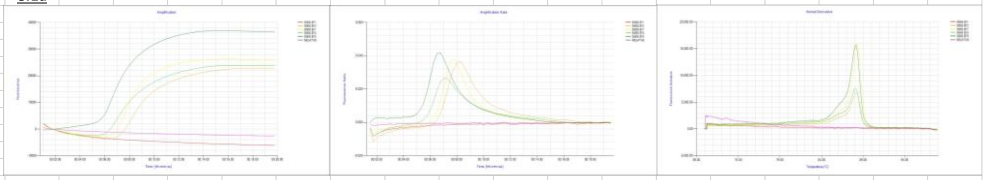

B **KHV**

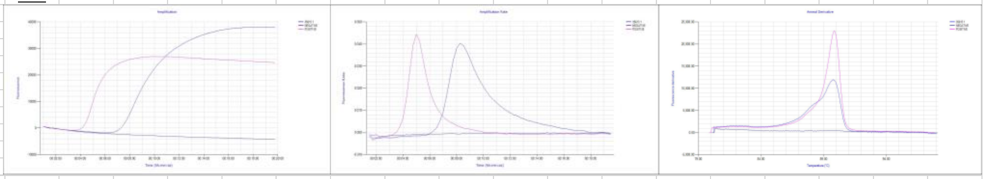

ef1a

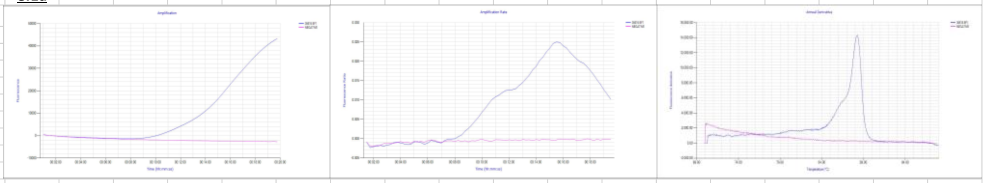

C **KHV**

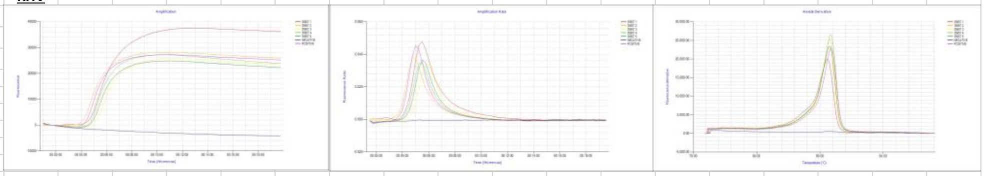

ef1a

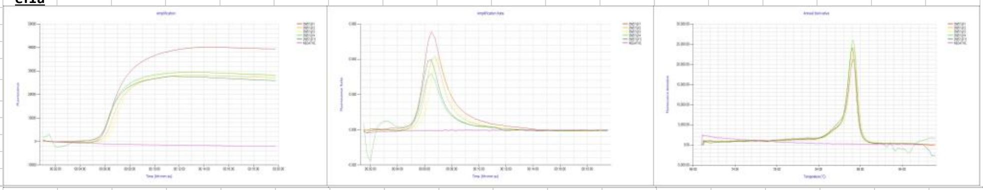

CEV

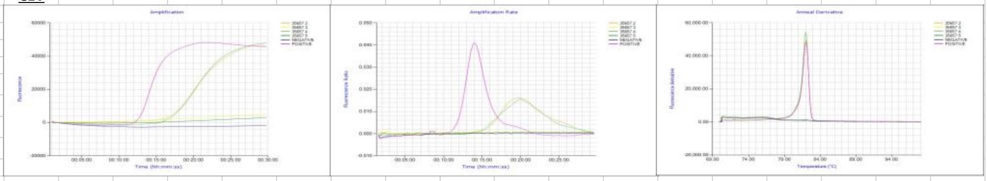

D **KHV**

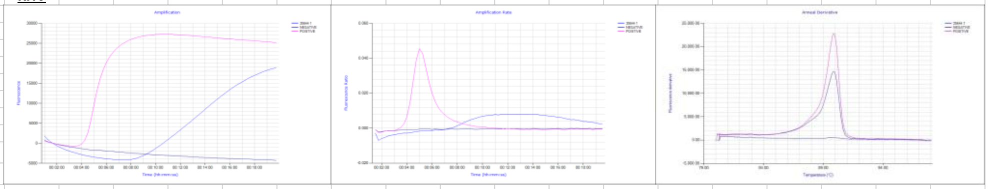

ef1a

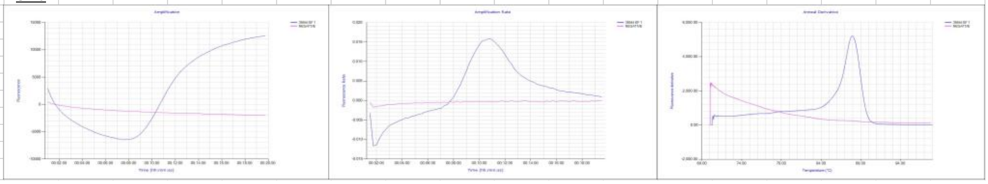

E **KHV**

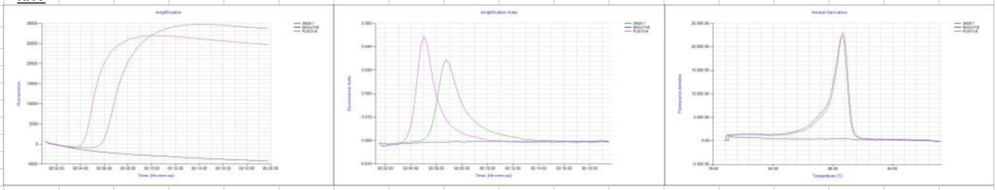

ef1a

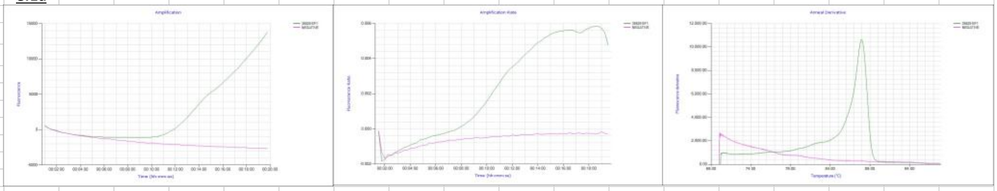

F **KHV**

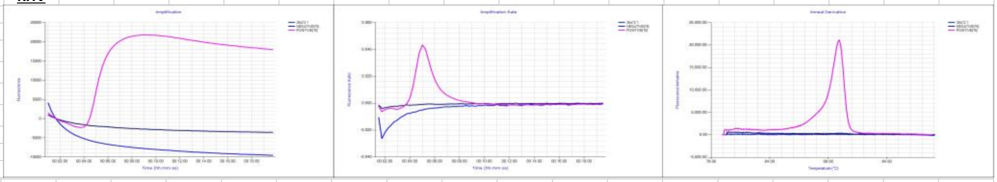

ef1a

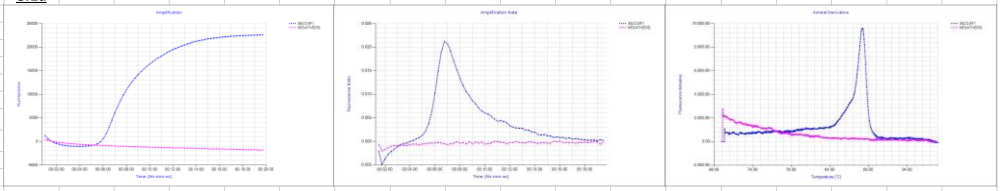

G **KHV**

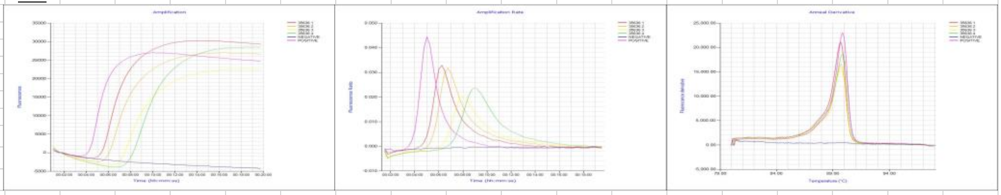

ef1a

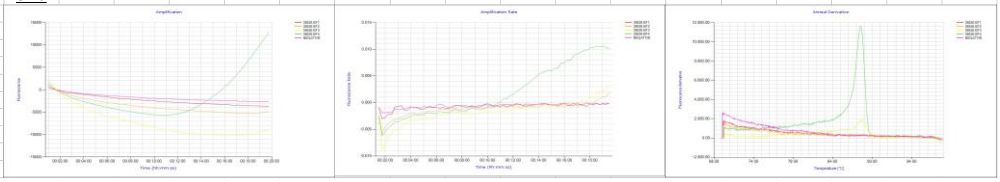

H **KHV**

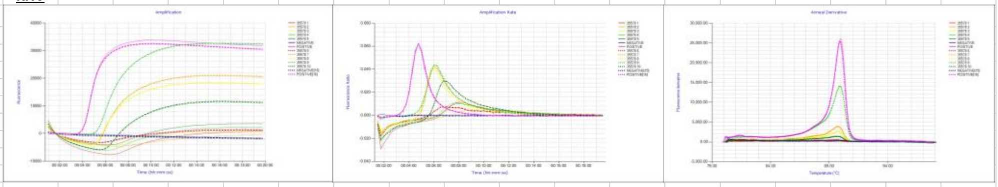

ef1a

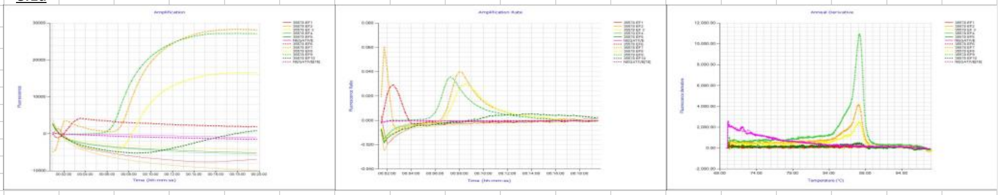

I **KHV**

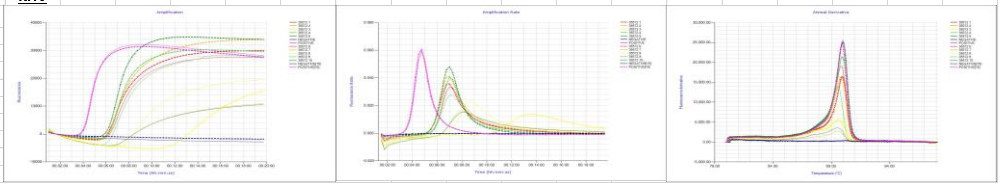

ef1a

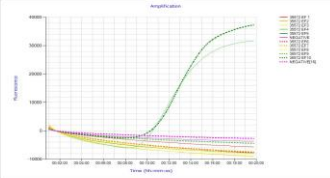

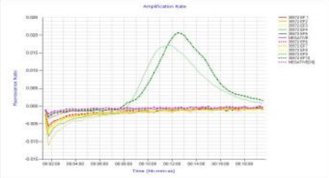

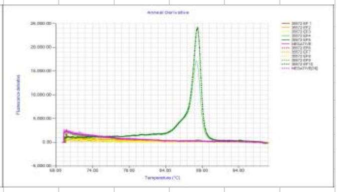

J **KHV**

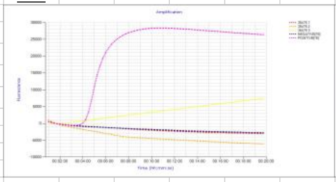

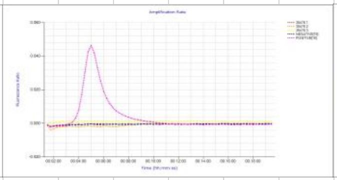

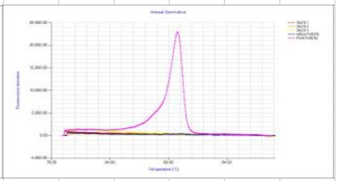

ef1a

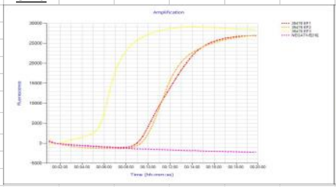

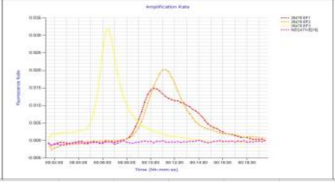

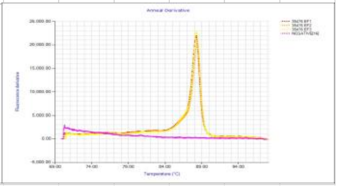

K **KHV**

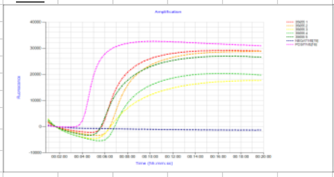

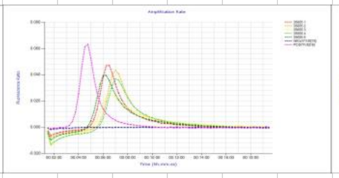

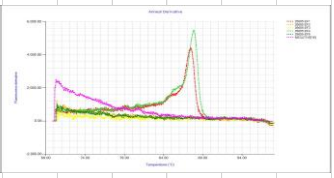

ef1a

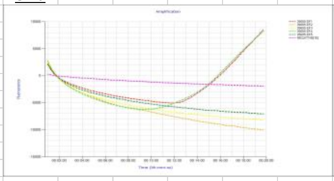

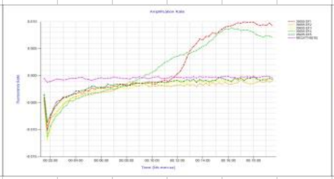

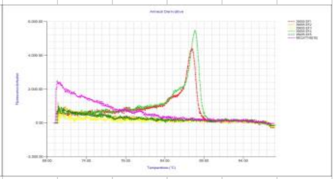

L **KHV**

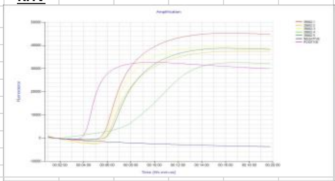

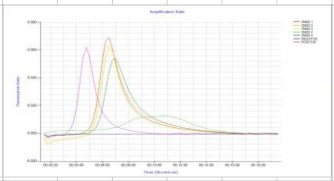

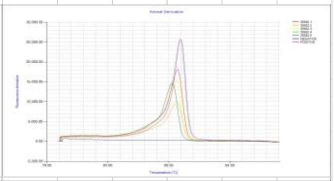

ef1a

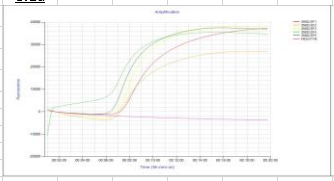

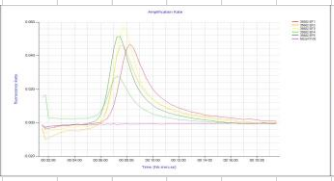

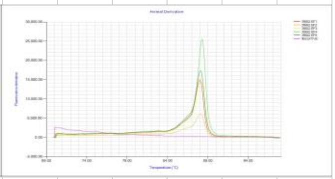

M **KHV**

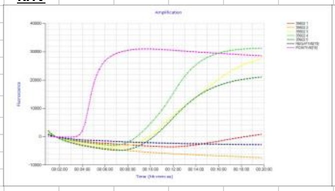

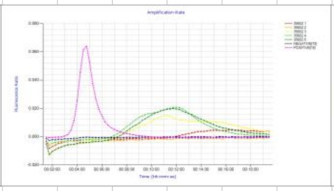

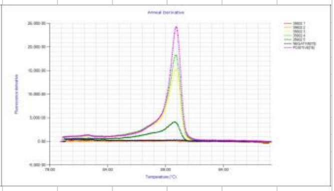

ef1a

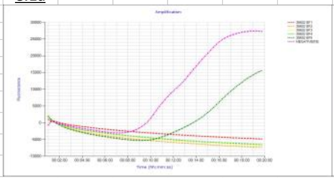

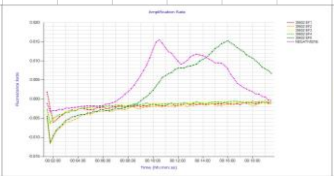

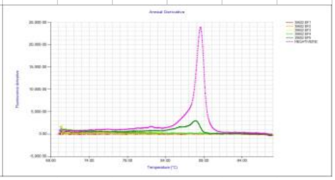

N **KHV**

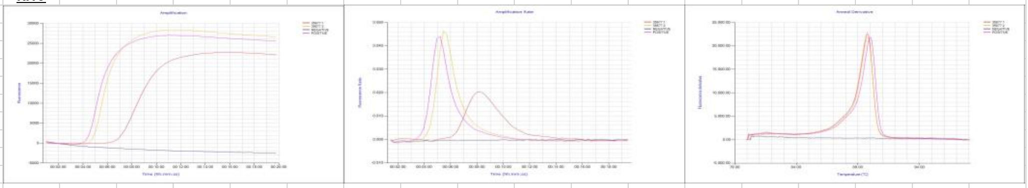

ef1a

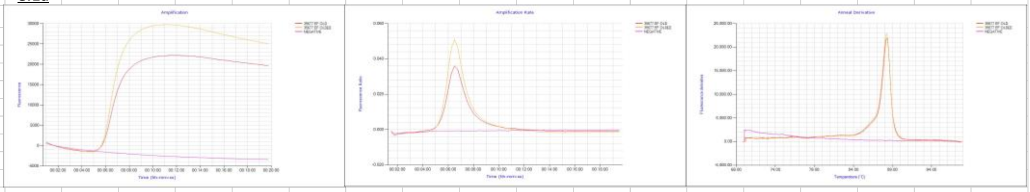

O **KHV**

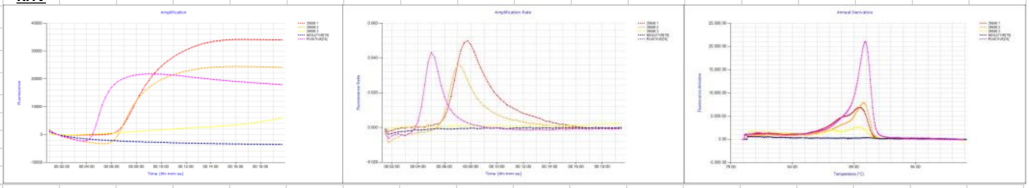

ef1a

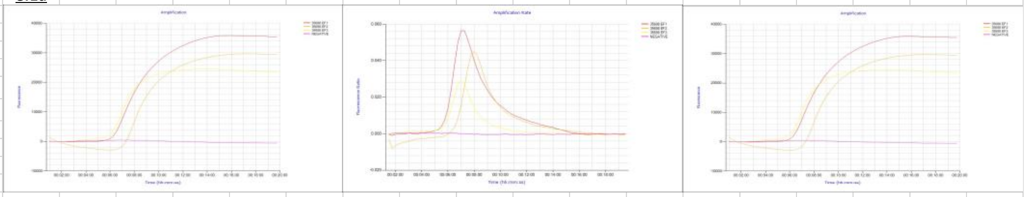

P **KHV**

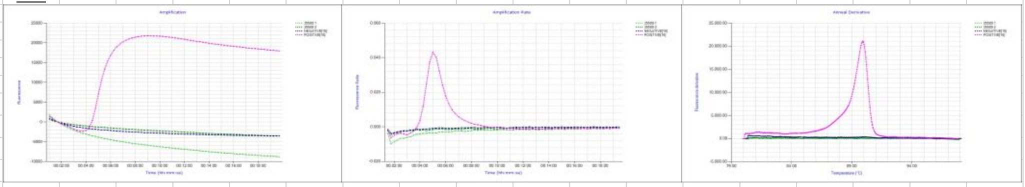

ef1a

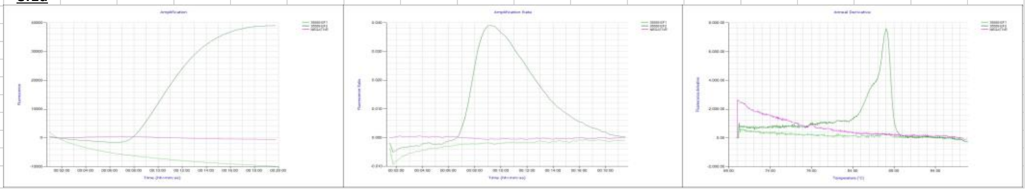

Supplement: Supplementary file 1 [file animals-11-00459-s001.zip › Figure S2.pdf]
